# Mathematical modelling of adult hippocampal neurogenesis: effects of altered stem cell dynamics on cell counts and bromodeoxyuridine-labelled cells

**DOI:** 10.1098/rsif.2014.0144

**Published:** 2014-05-06

**Authors:** Frederik Ziebell, Ana Martin-Villalba, Anna Marciniak-Czochra

**Affiliations:** 1 Institute of Applied Mathematics, University of Heidelberg, Heidelberg, Germany; 2 Interdisciplinary Center of Scientific Computing (IWR) and BIOQUANT, University of Heidelberg, Heidelberg, Germany; 3 German Cancer Research Center (DKFZ), Heidelberg, Germany

**Keywords:** neurogenesis, stem cells, mathematical modelling, ordinary differential equations

## Abstract

In the adult hippocampus, neurogenesis—the process of generating mature granule cells from adult neural stem cells—occurs throughout the entire lifetime. In order to investigate the involved regulatory mechanisms, knockout (KO) experiments, which modify the dynamic behaviour of this process, were conducted in the past. Evaluating these KOs is a non-trivial task owing to the complicated nature of the hippocampal neurogenic niche. In this study, we model neurogenesis as a multicompartmental system of ordinary differential equations based on experimental data. To analyse the results of KO experiments, we investigate how changes of cell properties, reflected by model parameters, influence the dynamics of cell counts and of the experimentally observed counts of cells labelled by the cell division marker bromodeoxyuridine (BrdU). We find that changing cell proliferation rates or the fraction of self-renewal, reflecting the balance between symmetric and asymmetric cell divisions, may result in multiple time phases in the response of the system, such as an initial increase in cell counts followed by a decrease. Furthermore, these phases may be qualitatively different in cells at different differentiation stages and even between mitotically labelled cells and all cells existing in the system.

## 1. Introduction

In the adult hippocampus, neurogenesis occurs in the subgranular zone of the dentate gyrus [1]. Here, individual stem cells are capable of generating astrocytes and neural progenitors. Recent data obtained from single cell level analysis demonstrate that stem cells perform four different types of events in order to produce progeny: symmetric divisions by dividing into two stem cells; two types of asymmetric divisions by either dividing into a stem cell and an astrocyte or a stem cell and a neural progenitor and astrogenic transformation; the direct conversion of a stem cell into an astrocyte [2]. Being born from stem cells, neural progenitors are capable of expanding their own pool by symmetric divisions and produce immature neurons called neuroblasts, which, in turn, mature to become neurons [2]. On a population level, it was shown that the number of stem cells, neural progenitors and immature neurons decreases during the ageing process [3,4] and alongside the number of newborn neurons depletes with time [5].

Over the past few years, knockout (KO) experiments have been used to study the neurogenic niche of the dentate gyrus, and the involved mechanisms governing stem cells' fate choice [2,6–10]. Evaluating the results of such experiments is a non-trivial task owing to the multifactorial nature of the neurogenesis process. These complex dynamics severely limit intuitive interpretation of experimental data and call for tools such as mathematical modelling and analysis. The aim of this study was to establish a basic model of adult hippocampal neurogenesis using previously published data. Because KO experiments had targeted stem cell compartments, a mathematical model describing the dynamics of cell counts for a given set of stem cell parameters provides a theoretical framework to identify the function of such KOs.

Mathematical and computational models have been applied before to study adult neurogenesis. Ashbourn *et al.* [11] provide a system of partial differential equations to model the migration of immature neurons from the subventricular zone along the rostral migratory stream to the olfactory bulb and investigate parameters that lead to biologically plausible solutions. Aimone *et al.* [12] model the functional integration of new neurons to the hippocampus as an artificial neural network. To the authors’ best knowledge, there exists no model addressing the cellular dynamics in the subgranular zone niche of the dentate gyrus.

Our proposed model of the adult hippocampus is a neurogenesis-adjusted modification of the model of haematopoiesis investigated by Marciniak-Czochra *et al.* [13] and Stiehl & Marciniak-Czochra [14]. Dynamics of hierarchical cell production systems, which maintain a continuous supply of differentiated functional cells to various parts of a living organism, have attracted the attention of biologists and mathematicians for many years in the context of blood cell production [15]. Besides common elements that can be found in all cell production systems, there are significant differences depending on the type of cells considered. To model the hierarchical structure of the system, we apply a system of ordinary differential equations (ODEs), each of which describes a discrete differentiation stage. In such models, the pace of commitment is dictated by successive divisions. However, in the case of neurogenesis, there are indications that stem cell differentiation also involves direct (continuous) transitions. Furthermore, neural stem cells are multipotent and generate, both, neurogenic progenitors and astrocytes. We develop a new model accounting for these observations, as presented in §2. Another important application of modelling is in the choice of regulatory mechanisms. Because we aim to model short-term dynamics of labelled cells, and there is no experimental evidence of feedback loops governing this process, we propose a linear model. This assumption stays in line with a parsimonious (reductionist) approach to modelling, in which comprehensive models are better understood in view of simpler models. It allows closed-form solutions to be obtained for the mathematical analysis of derivatives with respect to stem cell parameters.

Our study is organized as follows: in §2, we state an ODE model of adult hippocampal neurogenesis based on the experimental observations reviewed in the first paragraph of this introduction. Moreover, we introduce parameters that model the dynamics of neural stem and progenitor cells, namely the fraction of self-renewal, the proliferation rate and the division probability. In §3, we infer relations among these model parameters by deriving parameter conditions that account for the age-related decline in stem cell and progenitor counts as demonstrated by experimental data. Section 4 provides a mathematical analysis of the effects of a KO experiment. Because a stem-cell-targeting inducible KO spontaneously changes the dynamics of its target, we model such a KO by analysing the effects of alterations (calculating partial derivatives) with respect to the stem cell parameters proliferation rate, fraction of self-renewal and division probability on cell counts and on the number of bromodeoxyuridine (BrdU) incorporating cells. Section 5 contains parameter estimations and numerical investigations that could not be treated analytically and, in §6, we summarize and discuss our findings.

Basic notation: we occasionally write *x*(*t*; *p*) to emphasize the dependence of the solution *x*(*t*) of a differential equation on a parameter *p* and sgn(*a*) denotes the sign of a real number *a*.

## 2. Derivation of a multicompartmental model

Based on the experimental data outlined in §1, we assume a model of adult neurogenesis with five cellular compartments: stem cells (*c*_1_), (neural) progenitors (*c*_2_), neuroblasts (*c*_3_), mature neurons (*c*_4_) and astrocytes (*c*_5_):2.1
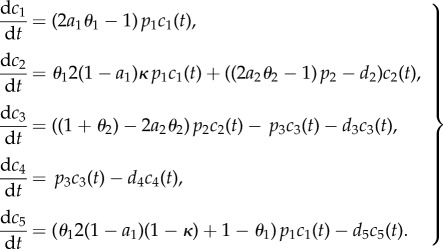


The model equations describe the following events: stem cells can either divide with probability *θ*_1_ or perform an astrocytic transformation with probability 1−*θ*_1_. The rate at which stem cells undergo these events is given by the parameter *p*_1_, called hereafter as the proliferation rate. The balance between symmetric self-renewal and asymmetric divisions is reflected by the fraction of self-renewal *a*_1_ ≥ 1/2, which is the probability that a daughter cell has the same fate as the mother cell. The relation
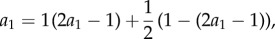
shows that 2*a*_1_ − 1 is the corresponding probability of a symmetric division and 1 − (2*a*_1_ − 1) = 2(1 − *a*_1_) the probability of an asymmetric division, because the fraction of daughter cells that continue as stem cells is 1 in a symmetric and 1/2 in an asymmetric division. It follows that the expected net change of the number of stem cells after one stem cell event (division or transformation) is given by
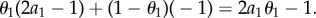


**Figure 1.**
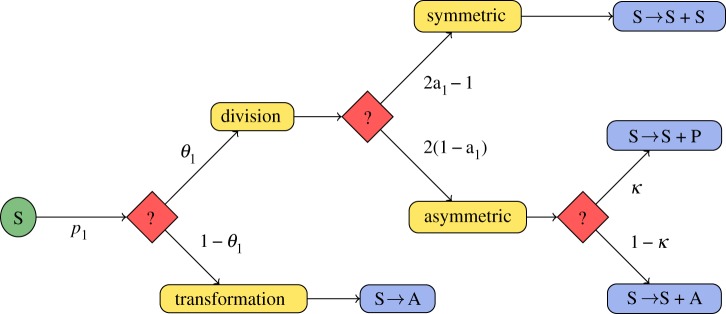
Proliferation diagram of a stem cell. Red nodes indicate events with stochastic outcome (e.g. division or transformation; symmetric or asymmetric division), blue nodes describe the outcome of particular events using chemical reaction notation (S, stem cell, P, neural progenitor, A, astrocyte). *θ*_1_ denotes the probability of stem cell division, *p*_1_ denotes the proliferation rate; *a*_1_ reflects the probability that a daughter cell has the same fate as its parent cell (self-renewal takes place) and *κ* is the probability that a neural progenitor is produced in an asymmetric division rather than an astrocyte.

Asymmetric cell divisions may lead to two types of differentiated cells. The non-stem daughter cell is assumed to be either a neural progenitor with probability *κ* or an astrocyte with probability 1 − *κ* (see figure 1 for the diagram showing possible scenarios followed by a stem cell).

For the proliferative capacity of progenitors, we again assume two possible modes of generating progeny: division, which occurs with probability *θ*_2_ or direct transformation to a neuroblast with probability 1 − *θ*_2_. Analogous to stem cells, progenitors have a proliferation rate *p*_2_ and a fraction of self-renewal *a*_2_. Neuroblasts mature by transforming into a neuron with rate *p*_3_. Furthermore, we assume that all cell types except stem cells are subjected to decay via apoptosis, modelled by the parameters *d_i_* corresponding to cellular compartment *i*.

The independent time variable *t* is used in two contexts. In §3, we analyse age-related properties of the neurogenesis system and use time for the adult age of the animal, i.e. the time point, *t* = 0, refers to the beginning of adult age, and the initial data consist of the number of cells present at *t* = 0 for each compartment. In §§4 and 5, we investigate the effect of altered stem cell dynamics on cell counts and on the number of BrdU-labelled cells in the framework of an inducible KO experiment, which imposes altered dynamics upon administration of a certain chemical. In this context, time is used as the time after the KO occurred, i.e. *t* = 0 refers to the start at which the neurogenesis system operates under the altered dynamics.

## 3. Decline of stem cell and progenitor counts

Because it was observed that the number of stem and progenitor cells declines with age [3], we first derive parameter conditions that account for this effect. The proof of the subsequent lemma is given in appendix A 1.

**Lemma 3.1.**

*The solutions* *c*_1_(*t*) *and* *c*_2_(*t*) of (2.1) *are monotonically decreasing for all* *t* ≥ *0 if and only if*
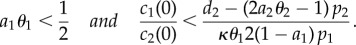

*Furthermore*
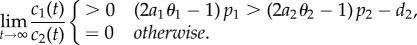

*Biological interpretation:* Lemma 3.1 states that the depletion of the stem cell pool takes place if and only if symmetric stem cell divisions, accompanied by a gain of stem cells, are less likely than astrocytic transformations with the resulting loss of the stem cell. The second part states that the ratio of the number of stem cells to the number of progenitors converges to zero, if the net depletion rate of stem cells is higher than the one of progenitors. Otherwise, it goes to a positive value. Furthermore, the positive steady state is attained monotonically, either increasing or decreasing. Interestingly, both behaviours have been observed experimentally. In reference [3], it was reported that the ratio of the number of stem cells to the number of progenitors is monotonically decreasing, whereas Jinno [16] reports an increasing progression. The discrepancy in both observations might thus come from different labelling paradigms and observations of different subpopulations.

In [3, electronic supplementary material, table S2], a time series for the age-related decline of the stem cell and progenitor count was obtained. Fitting these data to the solution of (2.1) indicates that the parameters of our model satisfy the relations (2*a*_2_*θ*_2_ − 1)*p*_2_ − *d*_2_ − (2*a*_1_*θ*_1_ − 1)*p*_1_ < 0 and *a*_1_*θ*_1_ < 1/2 (see §5.1). Hence, these data are consistent with the scenario in which the net depletion rate of progenitors is higher than the net depletion rate of stem cells. To summarize, we further make the following assumption in our subsequent mathematical analysis in order to remain consistent with the experimental data of reference [3]:

**Assumption 3.2.** The parameters of model (2.1) have the properties
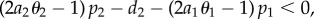
and
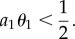

## 4. Mathematical analysis of altered stem cell parameters

### 4.1. Preliminaries

#### 4.1.1. The inducible knockout experiment

A gene-KO is a procedure that eliminates a certain gene (a DNA sequence encoding a protein) from an organism's DNA. Thus, the corresponding protein is not synthesized. In order to study the protein-driven regulatory mechanisms involved in the dynamics of adult neural stem cells, such KO experiments were conducted in the past. A particular version of KO experiments is the inducible KO: cells with a pre-marked gene-sequence react to the administration of a chemical that is injected in the animal, with the activation of a cutting enzyme that excises the marked sequence. Because it is possible that this cutting enzyme is only present in cells expressing a certain other protein, for instance, the adult stem cell characteristic protein nestin, one can selectively knock out the gene of interest in stem cells.

If the KO of a certain gene in the stem cell compartment results in a difference between KO and wild-type (non-KO) animals regarding the number of counted cells, the question arises as to which stem cell parameter was affected by the KO and caused the observed difference. To treat this question in a general way, we examine the effects of alterations of the stem cell parameters *a*_1_ (fraction of self-renewal), *θ*_1_ (division probability) and *p*_1_ (proliferation rate) on the number of existing cells and the number of cells labelled by BrdU, a chemical that is incorporated in the cells' DNA after cell division has taken place and during the stage of DNA synthesis.

#### 4.1.2. Modelling two experimental scenarios

**Figure 2.**
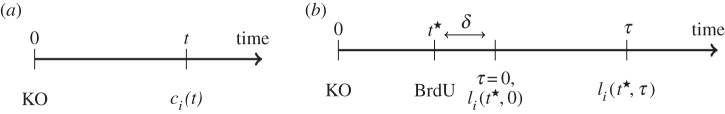
Graphical representation of the analysed knockout (KO) scenarios. (*a*) Cell counts *c_i_*(*t*) are evaluated at time *t* after the KO. (*b*) The number of BrdU-labelled cells is evaluated at time *τ* after the end of the labelling period (*τ* = 0) and BrdU was given at time 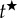 after KO.

We consider two scenarios related to KO experiments, for which we analyse the effect of altered stem cell parameters. *Scenario* (i)—(figure 2*a*): starting from a time point zero, which corresponds to the fixed age of the studied animal at which the KO is conducted, the number of cells of compartment *i* is analysed at *t* time units after time point zero. *Scenario* (ii)—(figure 2*b*): at time 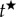 after the initial time point of the KO, BrdU is administered and is present in the organism for a duration *δ*, thus labelling DNA-synthesizing cells during that period. At *τ* time units after the labelling has ended, the number of BrdU-labelled cells 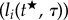 is examined.

To evaluate for both scenarios the effects of a change of a parameter 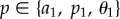 from a value 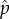 to a value 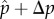 (Δ*p* > 0), we analyse the sign of the derivative 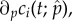 respectively, 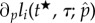 with respect to the parameter *p*. Thus, we assume that Δ*p* is so small, that
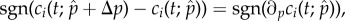
and
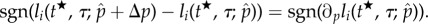


To model scenario (i), we use our model (2.1) together with initial data *c_i_*(0) corresponding to the number of cells of compartment *i*, which are present at the time point of the KO. For scenario (ii), the initial data are 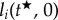, the number of cells that have incorporated BrdU at the end of the labelling period, where BrdU was given at time point 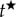 and the independent variable in this scenario is *τ*, the time that passed since the end of the labelling period.

#### 4.1.3. Initial data for BrdU-labelled cells

It is known from the theory of branching processes that in a model of proliferation in which particles (cells) have exponentially distributed lifetimes, mean counts of particles (cells) follow a system of ODEs [17, ch. 4]. Conversely, if the population of cells is described by a system of ODEs, then the interpretation is that the cells have exponentially distributed lifetimes. This is a simple and widely used model (see relevant discussion of the cell proliferation models in Kimmel & Axelrod [17]), despite the fact that it has been known that cell lifetime distributions are not exponentially distributed [18]. Thus, we use this relationship between ODEs and the exponential distribution in order to derive equations for the number of cells belonging to type *i* and have incorporated BrdU 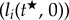.

Recall that BrdU is a chemical that is incorporated in cells after they performed a division and are in the stage of DNA synthesis. Thus, in order to be labelled by BrdU, a cell must be in S phase during the time interval of length *δ* in which BrdU is present in the animal. Assuming that the fraction of dividing cells equals the fraction of DNA-synthesizing cells during any time interval of fixed length, it follows that the number of cells that have incorporated BrdU at the end of the labelling period, starting with labelling at time 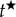, is given by4.1
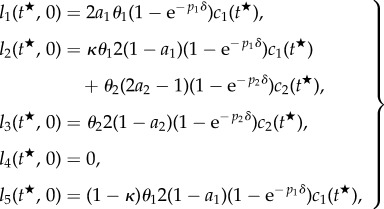
where 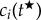 is the number of cells of compartment *i*, present at time 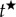 of BrdU injection. More specifically, a random variable 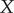, exponentially distributed with parameter *λ*, has the property4.2
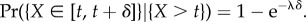
Thus, the fraction of stem cells performing a transformation or division during marker exposure is 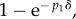 and a fraction *θ*_1_ of them divides with each division contributing, on average, 2*a*_1_ stem cells. Analogous considerations lead to the equations for all other cell compartments. Note that neurons are assumed to be the result of a transformation from neuroblasts rather than a division. Thus, there are no BrdU-labelled neurons right after the BrdU-labelling period has ended.

### 4.2. Effects of altered stem cell parameters

Based on the considerations of §4.1.3, we model the scenarios (i) and (ii) of §4.1.2 using the system of ODEs that follow. The equations for the quantities 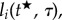 describing the number of BrdU-labelled cells of type *i* at *τ* time units after BrdU was started at time 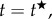 have been derived based on the assumption that labelled cells of type *i* follow the same dynamics as their corresponding compartment *c_i_*. Thus, the initial data for these labelled cells, 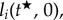 depend on 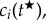 the number of cells present at the time point 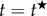 of BrdU injection.4.3
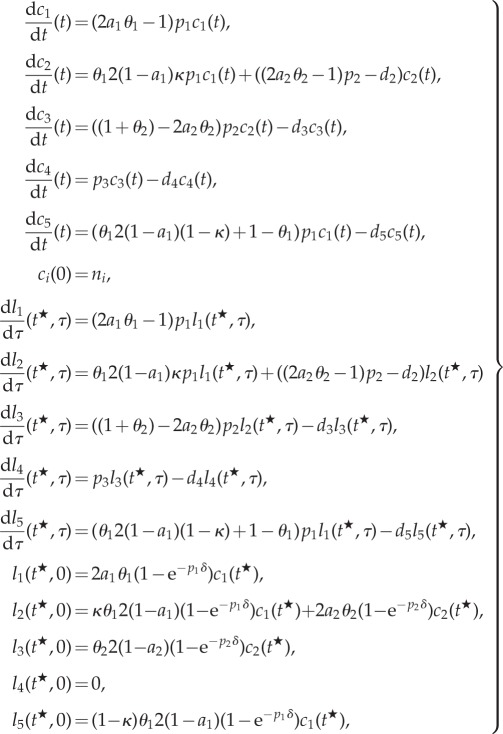
where *n_i_* is the number of cells of compartment *i* at the fixed age of the animal where the KO is induced. We mathematically analyse the derivatives of *c*_1_(*t*), *c*_2_(*t*) and *l*_1_(*t*, 0), *l*_2_(*t*, 0) and *l*_5_(*t*, 0) with respect to the parameter 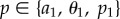, thus evaluating the implications of altered stem cell parameters on the number of existing stem cells and progenitors and on the number of BrdU-labelled stem cells, progenitors and astrocytes at the end of the labelling period. The parameter-derivatives of the number of neurons and BrdU-labelled neurons (*c*_4_(*t*) and *l_i_*(*t*,*τ*)) cannot be analysed in full generality for arbitrary *τ* and are thus investigated numerically in §5. For notational convenience, we define
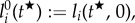
and denote this quantity as the number of BrdU incorporating cells of compartment *i*.

By a straightforward calculation (see appendix A 2), one obtains the identities4.4
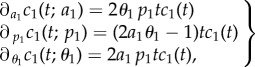
and4.5
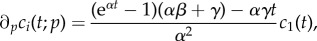
for 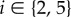 and 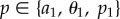, where *α*, *β* and *γ* depend on both, *i* and *p* (cf. table 2).

In order to evaluate (4.5), we need a technical lemma.

**Lemma 4.1.**

*For* *t ≥ 0, *α* < 0* *and* 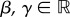, *consider the function*
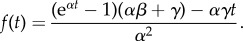

*It holds* *f*(0) = 0*,* 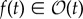 *and* *f* *has the following properties:*

(P1) *If β* > 0 and *γ* > 0,

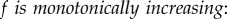

(P2) If *β* > 0 *and* *γ* < 0*, there exists a unique t*_0_ > 0 *such that for all t* > 0
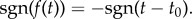

(P3) If *β* < 0 *and* *γ* > 0, *there exists a unique* *t*_0_ > 0 *such that for all* *t* > 0
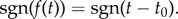

**Proof.**

Follows from evaluating 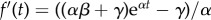 for every single case.

Furthermore, we introduce the notion of the *sign-sequence* of a real-valued function:

**Definition 4.2.**

(sign-sequence). Let 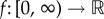 be a function. The sign-sequence *σ*(*f*) is defined as the sequence of distinct signs of *f*(*t*) that are encountered by traversing the domain of *f* from zero to infinity. For instance, 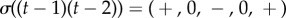.

#### 4.2.1. Altered fraction of self-renewal

Here, we describe the effect of increasing the fraction of self-renewal of stem cells. Recall that this fraction *a*_1_ is defined as the probability that a daughter cell, which resulted from a stem cell division, becomes a stem cell itself. Thus, increasing the fraction of self-renewal increases the proportion of symmetric stem cell divisions that give rise to two stem cells at the expense of asymmetric stem cell divisions.

**Lemma 4.3.**

*The solution of* (4.3) *satisfies*4.6
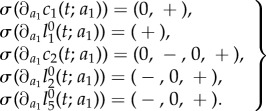

**Proof.**

From (4.3) and (4.4), it follows that4.7
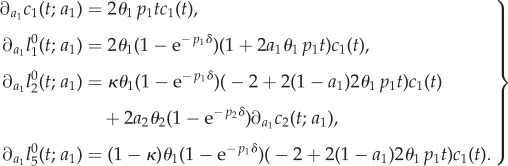

Thus, 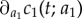 and 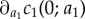 are positive for all positive *t* and 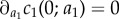. Consider now the quantity 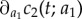. Assumption 3.2 together with table 2 implies that the first factor of 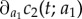 in (4.5) has the property (P3) in lemma 4.1. Consequently, 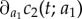 is negative on (0, *t*_0_) and positive on (*t*_0_, *∞*) for some positive on *t*_0_. From (4.7), we deduce that the same is true for 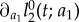 and 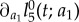, because the term − 2 + 2(1 − *a*_1_)2*θ*_1_*p*_1_*t* is negative for *t* = 0 and positive for sufficiently large *t*.▪

**Figure 3.**
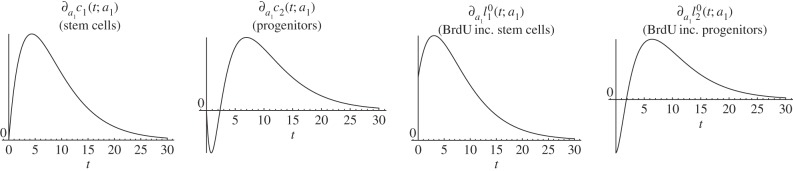
Simulated responses to an infinitesimal increase in stem cells fraction of self-renewal *a*_1_ of the number of stem cells (*c*_1_), progenitors (*c*_2_), BrdU incorporating stem cells 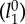 and BrdU incorporating progenitors 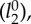 respectively, at time *t* after the increase. The parameter set of §5.2 was used.

*Biological interpretation*. Lemma 4.3 shows that increasing stem cells' self-renewal increases the stem cell count and the number of BrdU incorporating stem cells at any time point after the increase was performed. Conversely, this increased self-renewal initially decreases the progenitor count and the number of BrdU incorporating progenitors and astrocytes. But this decrease is reversed and turns into an increase after the initial period. Furthermore, the effect of altered self-renewal is instantaneous on the number of BrdU-labelled cells in the sense that the corresponding parameter-derivative is non-zero at time zero. Figure 3 depicts a simulation of the time-dependent responses of an increased fraction of self-renewal. This simulation is consistent with lemma 4.3.

The two-phase progression on progenitors and astrocytes can be explained intuitively as follows: the increased number of symmetric stem cell divisions at the expense of asymmetric divisions reduce the proportion of events at which progenitors are born. Thus, a decreased number of progenitors is observed initially. At the same time, the increased number of symmetric stem cell divisions, which result in an enlarged stem cell pool, benefits the progenitor count in the long run: although a reduced fraction of stem cells generates progenitors via asymmetric divisions, the increased number of stem cells dominates this effect, meaning that the total number of asymmetric stem cell divisions is elevated. The immediate effect on BrdU incorporating progenitors and astrocytes can be explained by the observation that changing a parameter that affects division instantaneously changes the output of the division and that the mitotic marker BrdU exactly labels this output. Thus, the number of cells labelled by BrdU is faster influenced by a parameter change than the actual cell count.

#### 4.2.2. Altered proliferation rate

The proliferation rate *p*_1_ is the rate at which stem cells undergo division or transformation events. Increasing this rate shortens the waiting time between successive events of a given stem cell.

**Lemma 4.4.**

*The solution of* (*4.3*) *satisfies*4.8
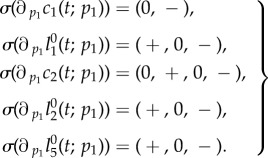

**Proof.**

Equations (4.3) and (4.4) imply4.9
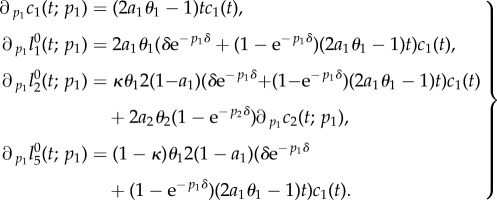

Because of the assumption 3.2, 2*a*_1_*θ*_1_ − 1 < 0. Thus, 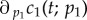 is negative on (0, *∞*) and 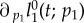 is positive on (0, *t*_0_) and negative on (*t*_0_, *∞*) for some positive *t*_0_. The same is true for 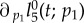, but with different *t*_0_. Consider now 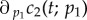. The first factor of 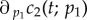 in (4.5) has the property (P2) in lemma 4.1. Hence, 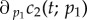 and 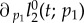 show the same qualitative progression as stated for 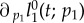. Analogous to the considerations of an altered fraction of self-renewal, the BrdU incorporating quantities 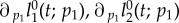 and 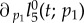 are positive at time zero, whereas 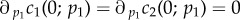.▪

**Figure 4.**
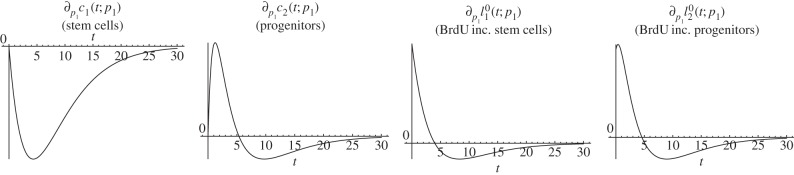
Simulated responses to an infinitesimal increase in stem cells proliferation rate *p*_1_ of the number of stem cells (*c*_1_), progenitors (*c*_2_), BrdU incorporating stem cells 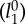 and BrdU incorporating progenitors 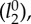 respectively, at time *t* after the increase. The parameter set of §5.2 was used.

*Biological interpretation*. Lemma 4.4 shows that an increased proliferation rate of stem cells decreases the stem cell count, whereas the number of BrdU incorporating stem cells initially increases and later on decreases. This effect can be explained by the observation that an increased proliferation rate causes more stem cell divisions over any given time interval, resulting initially in more BrdU incorporating stem cells. Furthermore, the increased proliferation rate also causes a higher rate of astrocytic transformations, the events responsible for the depletion of the stem cell pool. As time progresses, the increased decay rate of stem cells compensates for the higher proportion of BrdU incorporating cells and results thus in a net decrease of labelled stem cells. See figure 4 for a corresponding simulation, which is consistent with lemma 4.4. It is not surprising that the number of progenitors and BrdU-labelled progenitors and astrocytes display the same qualitative trend as labelled stem cells, because these quantities also depend on stem cell divisions.

#### 4.2.3. Altered division probability

The division probability *θ*_1_ of a stem cell is the probability that the next event a stem cell undergoes is a division rather than a transformation. Consequently, increasing the division probability causes more division and fewer transformation events.

**Lemma 4.5.**

*The solution of* (*4.3*) *satisfies*4.10
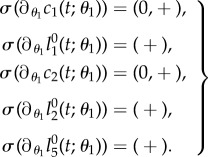

**Proof.**

It holds4.11
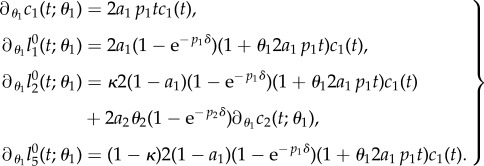

Thus, 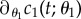, 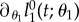 and 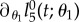 are positive for positive *t*. Furthermore, the first factor of 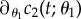 in (4.5) has the property (P1) in lemma 4.1. Hence, 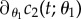 and 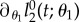 are also positive for positive *t*.▪

**Figure 5.**
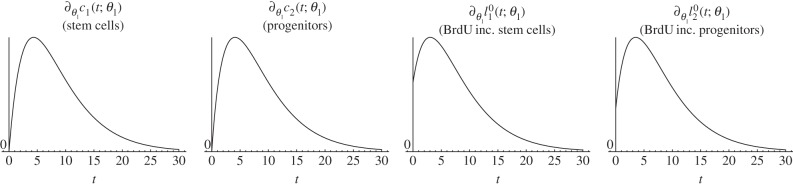
Simulated responses to an infinitesimal increase in stem cells division probability *θ*_1_ of the number of stem cells (*c*_1_), progenitors (*c*_2_), BrdU incorporating stem cells 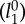 and BrdU incorporating progenitors 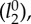 respectively, at time *t* after the increase. The parameter set of §5.2 was used.

*Biological interpretation*. From lemma 4.5, we conclude that increasing the stem cell division probability causes an increase in cell counts and in the number of BrdU incorporating cells for all considered compartments. There is no two-phase progression displaying increased or decreased cell numbers in the distinct phases after the change in stem cell dynamics. The effect on the progenitor count is qualitatively the same as on the stem cell count; the same holds true for BrdU incorporating stem cells and progenitors. Figure 5 illustrates a corresponding simulation of the discussed quantities.

### 4.3. Final remarks

The above considerations show that all derivatives of the quantities *c*_1_, *c*_2_, 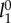, 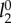 and 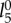 with respect to the stem cell parameters fraction of self-renewal, proliferation rate and division probability are products of the exponentially decreasing function *c*_1_(*t*) and a factor that can be bounded by an affine linear function of *t*. Thus, the effects of an altered stem cell parameter on the number of existing cells and the number of BrdU incorporating cells weakens with time owing to the decline of the stem cell compartment. The effect of altered stem cell parameters on the number of existing astrocytes, 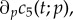 could not be analysed owing to no available data on the sign of *αβ* + *γ* with *α*, *β* and *γ* as stated in table 2.

The sign sequences of the parameter-derivatives of the five quantities stem cell count, progenitor count and BrdU incorporating stem cells, progenitors and astrocytes with respect to the three considered parameters *a*_1_, *p*_1_ and *θ*_1_ are summarized in table 1.

**Table 1.** Time-dependent responses of stem cells (*c*_1_), BrdU incorporating stem cells 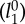, progenitors (*c*_2_), BrdU incorporating progenitors 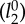 and BrdU incorporating astrocytes 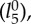 respectively, to an infinitesimal increase of the respective parameter *p*.

*p*	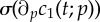 (stem cells)	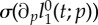 (BrdU inc. stem cells)	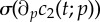 (progenitors)	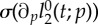 (BrdU inc. progenitors)	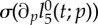 (BrdU inc. astrocytes)
*a*_1_	(0, +)	(+)	(0, −, 0, +)	(−, 0, +)	(−, 0, +)
*p*_1_	(0, −)	(+, 0, −)	(0, +, 0, −)	(+, 0, −)	(+, 0, −)
*θ*_1_	(0, +)	(+)	(0, +)	(+)	(+)

## 5. Numerical investigations

### 5.1. Parameter estimations

**Figure 6.**
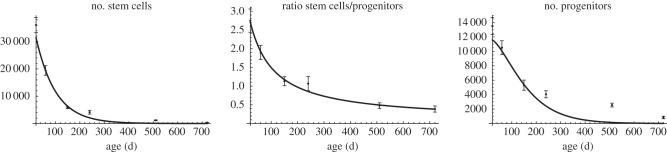
Fitting model (2.1) to the number of stem cells and the ratio of the number of stem cells to the number of progenitors in [3, electronic supplementary material, table S2] results in a poor agreement with the number of progenitors.

**Figure 7.**
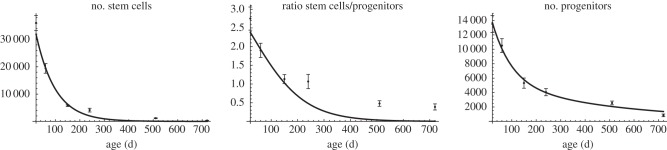
Fitting model (2.1) to the number of stem cells and the number of progenitors in [3, electronic supplementary material, table S2] results in a poor agreement with the ratio of the number of stem cells to the number of progenitors.

Our neurogenesis model (2.1) involves 18 parameters, including five parameters for the initial data of each compartment. We first analyse the parameter region that is consistent with the data presented in [3, electronic supplementary material, table S2]. For this purpose, we simultaneously fitted the analytical solution of (2.1) either for *c*_1_ and *c*_1_/*c*_2_ (the number of stem cells and the ratio of the number of stem cells to the number of progenitors), depicted in figure 6, or for *c*_1_ and *c*_2_ (the number of stem cells and the number of progenitors), shown in figure 7. Fitting was performed by using the *NonlinearModelFit* procedure of Mathematica v. 9 to numerically minimize the sum of squared residuals weighted by the inverse-square of the data points standard error of the mean as documented in the Mathematica reference for fitting data involving measurement errors. For numerical minimization, the random search method was chosen, which resulted in the highest *R*^2^ value among all available minimization methods.

Fitting the solution for *c*_1_(*t*) and 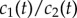 of (2.1) to the stated data results in the values5.1
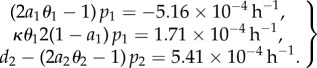


Although this parameter set is in good agreement with the data of reference [3] for the age-related decay of the stem cell compartment and the dynamics on the ratio of the number of stem cells and the number of progenitors, the fit to the decay of the progenitor compartment is not good (figure 6). Conversely, fitting the solution for *c*_1_(*t*) and *c*_2_(*t*) to the data of [3, electronic supplementary material, table S2], displays a good agreement of the stem cell and the progenitor compartment and a poor agreement of their ratio (figure 7).

It appears that the decay of the stem cell compartment involves a saturation effect for late time points, which cannot be reproduced by our linear model. More precisely, the solution of (2.1) for the number of stem cells (*c*_1_) is an exponentially declining curve and fitting this curve to the data estimates five stem cells remaining at 2 years of age, whereas this number was measured to be 320. We hypothesize that this saturation is caused by either a feedback mechanism on stem cells that induces their quiescence with increasing age or by the existence of a mixture of two populations with one population performing adult neurogenesis and a quiescent one. Moreover, physiological conditions of *a*_1_, *p*_1_, *θ*_1_ and *κ*, i.e. *a*_1_ *≈* 0.5, 1/*p*_1_ < 2 years, *θ*_1_ > 0.1 and *κ* > 0.5 contradict the restrictions imposed by (5.1), potentially because of the missing saturation effect that cannot be explained with our model. Thus, our current model indicates that there are some novel aspects in adult neurogenesis required to explain obtained experimental data such as this saturation effect. The proposed explanation of how saturation could be achieved should be the subject of future experimental validation.

### 5.2. Simulations

The mathematical analysis conducted in §4 depends on assumption 3.2. Because the purpose of this section is to extend our analysis to the effects of altered stem cell parameters on the number of neurons (*c*_4_) and the number of BrdU-labelled cells (*l_i_*(*t*,*τ*)) for *τ* > 0, we use a parameter set satisfying assumption 3.2 for our numerical investigations. Unless otherwise stated, we set *a*_1_ = 0.55, *θ*_1_ = 0.7, *p*_1_ = 1, *κ* = 0.6, *a*_2_ = 0.7, *θ*_2_ = 0.4, *p*_2_ = 2.5, *d*_2_ = 0.1, *p*_3_ = 1.5, *d*_3_ = 0.4, 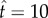, *d*_4_ = 0.05, *d*_5_ = 0.05, *c*_1_(0) = 10 000, *c*_2_(0) = 5000, *c*_3_(0) = 15 000, *c*_4_(0) = 350 000, *c*_5_(0) = 100 000 to numerically solve (2.1) by using the *NDSolve* framework of the Mathematica. Note that the stated parameter set does not include any time units, because a choice of physiological parameters was not feasible as stated in §5.1. Thus, there are no time units in any figure using this parameter set.

At first, we investigate the effect of altered stem cell parameters on the number of existing neurons at time *t* after the change of stem cell dynamics, i.e. 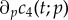 for 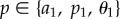.

**Figure 8.**
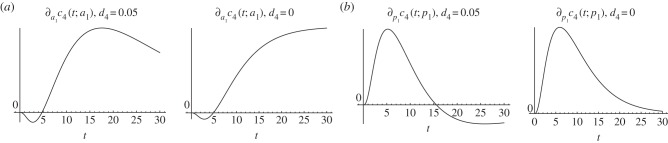
Responses to an infinitesimal increase in stem cells fraction of self-renewal and proliferation rate, respectively, on the number of neurons. (*a*) Effect of an increased fraction of self-renewal with and without death of neurons, respectively. (*b*) Effect of an increased proliferation rate with and without death of neurons, respectively.

An increase of *a*_1_, which increases the proportion of symmetric stem cell divisions at the expense of asymmetric divisions, displays the same qualitative progression on the number of neurons as the effect of an increase in *a*_1_ on the number of progenitors: initially, the neuron count is decreased, followed by an always ongoing period displaying an increase, but our simulations suggest that the magnitude of the increase weakens with time, i.e. 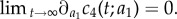 Interestingly, the existence of this weakening depends on the decay rate of neurons: if there is no decay (*d*_4_ = 0), then we find that 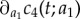 converges to a positive value (figure 8*a*). Hence, the magnitude of the increase is not declining with time, if neurons are not allowed to decay.

Increasing the proliferation rate *p*_1_ of stem cells also affects the number of neurons in the same way as the number of progenitors, so that in the initial period, the neuron count is increased, in a subsequent period, the neuron count is decreased and the magnitude of the effect declines with time. Interestingly, if we assume, in this case, that neurons do not decay, the two-phase progression is lost and, the number of neurons is always increased, but the magnitude of the increase still weakens with time (figure 8*b*).

Increasing the probability of stem cell divisions *θ*_1_ displays an increase in the number of neurons. If *d*_4_ > 0, our simulations indicate that the magnitude of the increase converges to zero and if *d*_4_ = 0 that it converges to a positive value (no figure is shown).

Our analysis indicates that the effect of altered stem cell dynamics on the number of neurons depends on the death rate of neurons. Thus, investigating whether neurons have the ability to decay and if so determining their decay rate is vital in order to understand the short- and long-term impact of altered stem cell dynamics on the number of neurons in the dentate gyrus.

**Figure 9.**
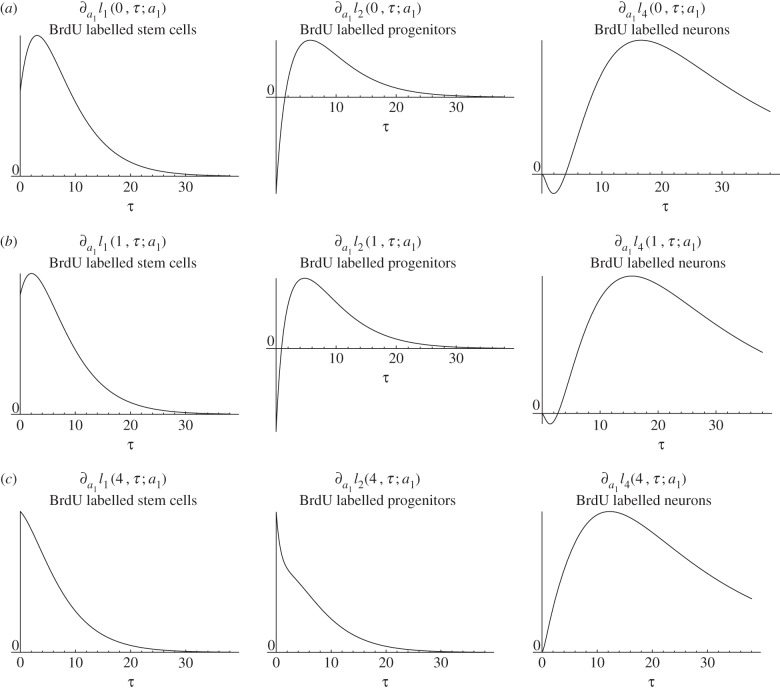
Effect of an infinitesimally increased stem cell self-renewal on BrdU-labelled stem cells, progenitors and neurons at time *τ* after BrdU-labelling, where BrdU was given at time *t* = 0 (*a*), *t* = 1 (*b*) and *t* = 4 (*c*).

Next, we turn our investigations to the quantity 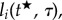 the number of BrdU-labelled cells of cellular compartment *i*, where BrdU was applied at a particular time point 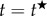 and *t* = 0 corresponds to the time point of the KO. The effect of an increase of stem cells fraction of self-renewal on BrdU-labelled cells depends on the time point 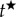. If BrdU was given directly after the fraction of self-renewal was increased, i.e. 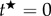, the number of BrdU-labelled progenitors *l*_2_(0,*τ*) shows a two-phase progression with an initial decrease and subsequent increase. The same holds true for the number of BrdU-labelled neurons *l*_4_(0, *τ*) (figure 9, top row). As the time 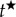 between the change of dynamics and the BrdU administration increases, the first phase that shows a decrease in the number of progenitors and neurons becomes shorter (figure 9, middle row) until there remains only a one-phase progression, with increased numbers of BrdU-labelled progenitors and neurons at every time point *τ* after BrdU was given (figure 9, bottom row).

**Figure 10.**
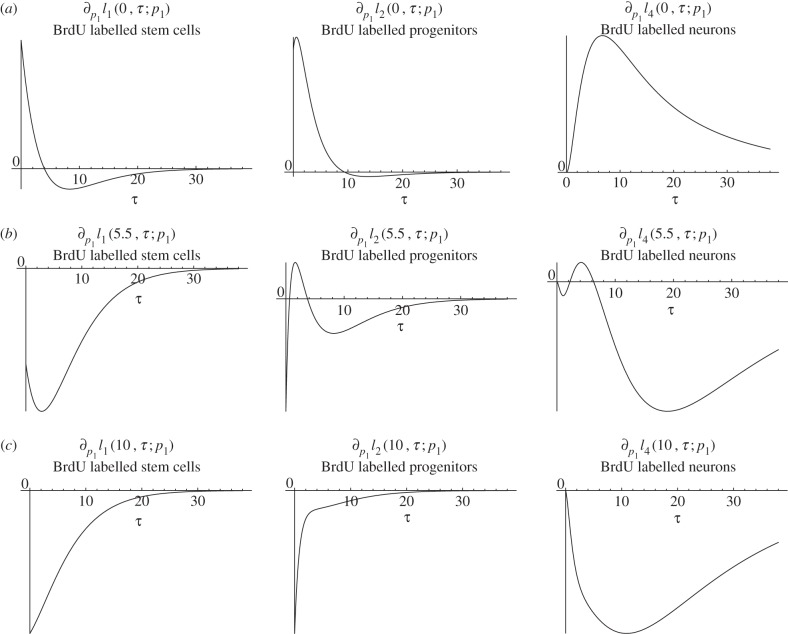
Effect of an infinitesimally increased stem cell proliferation rate on BrdU-labelled stem cells, progenitors and neurons at time *τ* after BrdU-labelling, where BrdU was given at time *t* = 0 (*a*), *t* = 5.5 (*b*) and *t* = 10 (*c*).

Similar to an increased fraction of self-renewal, the effect of an increased stem cell proliferation rate depends on the time 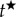 between the change of dynamics and the BrdU administration. If 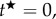 then BrdU-labelled progenitors and neurons display a two-phase progression with increased numbers in the initial phase and decreased numbers subsequently (figure 10, top row). Interestingly, the two-phase progression after BrdU-labelling gains an additional phase as 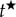 increases, displaying now an initial decrease, a subsequent increase and again later on a decrease in the number of BrdU-labelled progenitors and neurons (figure 10, middle row). Increasing 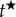 further, the three-phase progression is lost, and then the number of labelled progenitors and neurons is decreased at any time *τ* after BrdU was given (figure 10, bottom row).

## 6. Discussion and conclusion

We have established a mathematical model of adult hippocampal neurogenesis based on experimental data. Although we consider a basic model not accounting for any feedback mechanisms or a spatial component, we demonstrate that modifying the dynamics of adult neural stem cells, which corresponds to inducing a stem-cell-targeting KO, exhibits a rich variety of effects owing to the high complexity of the hippocampal neurogenic niche.

Our investigation shows that observed differences in cell numbers owing to altered stem cell dynamics depend not only on the alteration that was induced by a particular KO, but also on the time at which cell counts were measured. Therefore, it is necessary to perform measurements at multiple time points in order to draw conclusions from KO experiments. Moreover, we find that cell numbers of cells at different differentiation stages may respond qualitatively different to altered stem cell dynamics. Additionally, this response may also be different between the total number of cells and the number of BrdU-labelled cells of a given cellular compartment and also depends on the time point of BrdU administration after the KO. Thus, labelling cells with BrdU does not generate a subset of the neurogenesis system that is sufficient to perform an analysis of the impact of altered stem cell dynamics. The reason for this is that changing stem cell dynamics influences either the number of cell divisions or the ratio of symmetric to asymmetric divisions, which, in turn, affects the initial distribution of cell types among all BrdU-labelled cells. Furthermore, our reasoning proves that under the assumptions of our model, the effect of altered stem cell dynamics declines as time passes and that this decline is a result of the depletion of the stem cell pool. Evaluating differences in cell counts at late time points after a KO can therefore be used to test the notion of a declining stem cell population.

With the help of our study, the available data on KO experiments can be revisited in a new theoretical context: in reference [6], a KO of the gene Notch1 is analysed at multiple time-points after the KO. The counts of stem cells, neural progenitors and mature neurons decreased at late time points, whereas they slightly decreased or remain unchanged at earlier time points [6] (figure 2). The authors conclude that Notch1 KO results in a decreased self-renewal of stem cells. Moreover, they infer from neurosphere assays that neural progenitors also exhibit decreased self-renewal in the case of Notch1 KO. Their reasoning and data are consistent with our theoretical findings: decreasing the self-renewal of stem cells (cf. §4.2.1) results in decreased counts of stem cells at any time after the KO, whereas the progenitor cell counts exhibit an initial growth followed by a subsequent decline. Additionally, decreased self-renewal of progenitors is observed. This simultaneous decrease can compensate the initial increase of progenitor numbers caused by the decreased self-renewal of stem cells such that the number of progenitors is also decreased at any time after the KO. In another study of Furutachi *et al.* [8], it is reported that p57 is responsible for the quiescence of adult neural stem cells in the hippocampus: a KO of p57 resulted in a decrease of the number of BrdU-label-retaining stem cells at 60 days after the KO (fig. 2*k* of [8]), whereas the progenitor count initially displayed an increase and a subsequent decrease (figs 4*b* and 6*f* of [8]). In the context of our model, the claim of the authors corresponds to the statement that the KO of p57 causes an increase of the stem cell proliferation rate *p*_1_. As stated in §4.2.2, an increase of *p*_1_ results in an initial increase and subsequent decrease in the number of progenitors. Thus, the data presented and the authors' explanation that p57 KO decreases the quiescence of stem cells is consistent with the proposed mathematical model.

Our modelling approach qualitatively describes the effects of altered stem cell dynamics on cell counts and on BrdU-labelled cells. In order to calculate the magnitude of these effects and the time points at which positive and negative effects are to be expected, an estimation of model parameters accompanied by a possible data-driven modification of the proposed model has to be performed. Thus, a thorough characterization of wild-type neurogenesis is needed in order to evaluate KO experiments within a mathematical framework and to draw conclusions about the underlying dynamics of adult hippocampal neurogenesis.

## Appendix A

### A.1. Proof of lemma 3.1

It holds  if and only if  To analyse the decay conditions of the progenitor compartment *c*_2_, we introduce the abbreviations

A1

First, let us note that *p* < 0 and *r* > 0 is a necessary though not sufficient condition for . Furthermore, it can be seen that the quantity  satisfies a Riccati differential equation:

A2

Because *q* > 0, the right-hand side of (A 2) describes a downward opening parabola with roots 0 and  If *p* + *r* > 0, the state  is globally asymptotically stable. Otherwise, 0 is globally asymptotically stable. In both cases, the steady state is approached monotonically. Furthermore, *p* < 0 and *q*, *r* > 0 imply

A.3

and hence

A4

Note from (2.1) that  holds if and only if

Taken together with (A 4), we see that  for all *t* ≥ 0 if and only if

A5

▪

### A.2. Derivatives with respect to parameters

Here, we derive the identities (4.4)–(4.5) and the parameter-dependent values for *α*, *β* and *γ* in the latter equation. For this purpose, we need lemma A.1, which we prove at the end of the section. Lemma A.1. Let , *f, g* *real-valued, non-zero functions and consider the system*

A6

*together with non-zero initial conditions* *x*(0) *and* *y*(0), *which are independent of* *p*. *The solution then satisfies*

A7

*and*

A8

*where*

A9

In order to obtain (4.4) and (4.5), we apply lemma A.1 to (4.3) with *x* = *c*_1_,  and . Thus, the derivatives appearing in (A 9) have to be understood as partial derivatives with respect to the considered parameter . It follows that

If *y* = *c*_2_, we have

and if *y* = *c*_4_, it holds

The explicit values of *α*, *β* and *γ* appearing in (A 9) are summarized in table 2.

**Table 2.** Coefficients of  for

*p*	*i* = 2	*i* = 5
*a*_1_		
*p*_1_		
*θ*_1_		

Proof of lemma A.1. Define  and  . Because *x*(0) and *y*(0) are independent of *p*, we have

A10

and it follows from symmetry of second partial derivatives that

Hence,

The assumption *x*(0) *≠* 0 leads to  by (A 10) and solving the above differential equation yields

Furthermore,

and

imply

with *α*, *β* and *γ* as stated in (A 9), because a differential equation of the form

has the solution

▪
